# Enhancement of D-lactic acid production from a mixed glucose and xylose substrate by the *Escherichia coli* strain JH15 devoid of the glucose effect

**DOI:** 10.1186/s12896-016-0248-y

**Published:** 2016-02-19

**Authors:** Hongying Lu, Xiao Zhao, Yongze Wang, Xiaoren Ding, Jinhua Wang, Erin Garza, Ryan Manow, Andrew Iverson, Shengde Zhou

**Affiliations:** Hubei Provincial Cooperative Innovation Center of Industrial Fermentation, Key Laboratory of Fermentation Engineering (Ministry of Education), College of Bioengineering, Hubei University of Technology, Wuhan, 430068 P. R. China; Department of Biological Sciences, Northern Illinois University, DeKalb, IL 60115 USA; William Rainey Harper College, Palatine, IL 60142 USA

**Keywords:** Catabolite repression, D-lactic acid, *E. coli*, Glucose effect, *ptsG*, PLA, Xylose fermentation

## Abstract

**Background:**

A thermal tolerant stereo-complex poly-lactic acid (SC-PLA) can be made by mixing Poly-D-lactic acid (PDLA) and poly-L-lactic acid (PLLA) at a defined ratio. This environmentally friendly biodegradable polymer could replace traditional recalcitrant petroleum-based plastics. To achieve this goal, however, it is imperative to produce optically pure lactic acid isomers using a cost-effective substrate such as cellulosic biomass. The roadblock of this process is that: 1) xylose derived from cellulosic biomass is un-fermentable by most lactic acid bacteria; 2) the glucose effect results in delayed and incomplete xylose fermentation. An alternative strain devoid of the glucose effect is needed to co-utilize both glucose and xylose for improved D-lactic acid production using a cellulosic biomass substrate.

**Results:**

A previously engineered L-lactic acid *Escherichia coli* strain, WL204 (Δ*frdBC* Δ*ldhA* Δ*ackA* Δ*pflB* Δ*pdhR ::pflBp6-acEF-lpd* Δ*mgsA* Δ*adhE*, Δ*ldhA*::*ldhL*), was reengineered for production of D-lactic acid, by replacing the recombinant L-lactate dehydrogenase gene (*ldhL*) with a D-lactate dehydrogenase gene (*ldhA*). The glucose effect (catabolite repression) of the resulting strain, JH13, was eliminated by deletion of the *ptsG* gene which encodes for IIBC^glc^ (a PTS enzyme for glucose transport). The derived strain, JH14, was metabolically evolved through serial transfers in screw-cap tubes containing glucose. The evolved strain, JH15, regained improved anaerobic cell growth using glucose. In fermentations using a mixture of glucose (50 g L^−1^) and xylose (50 g L^−1^), JH15 co-utilized both glucose and xylose, achieving an average sugar consumption rate of 1.04 g L^−1^h^−1^, a D-lactic acid titer of 83 g L^−1^, and a productivity of 0.86 g L^−1^ h^−1^. This result represents a 46 % improved sugar consumption rate, a 26 % increased D-lactic acid titer, and a 48 % enhanced productivity, compared to that achieved by JH13.

**Conclusions:**

These results demonstrated that JH15 has the potential for fermentative production of D-lactic acid using cellulosic biomass derived substrates, which contain a mixture of C6 and C5 sugars.

## Background

Blending poly D-lactic acid (PDLA) and poly L-lactic acid (PLLA) with a determined ratio creates a stereo-complex of poly-lactic acid (SC-PLA) with an increased thermal tolerance up to 230 °C [10, 26]. This SC-PLA could expand PLA applications to agriculture-oriented and/or motor vehicle-associated plastics [6, 10, 25]. Manufacturing of this SC-PLA requires cost-effective production of optically pure D- and L-lactic acid isomers [1, 9].

Glucose derived from corn starch is currently used for industrial L-lactic acid fermentation. This process, however, competes with food resources. An alternative non-food substrate such as cellulosic biomass should be used for cost-effective lactic acid fermentation [9, 14, 15, 20]. The roadblock of this effort is that most lactic acid bacteria are unable to ferment xylose, a major constituent in the sugar stream of cellulosic biomass [14, 15]. Other strains, such as *Escherichia coli,* with the ability to utilize hexose and pentose sugars have been engineered for the production of D-lactic acid [2–4, 12, 16–19, 21,27–29, 32, 33]. Nevertheless, most studies reported the D-lactic acid production from glucose and/or sucrose by an engineered *E. coli* strain, with a titer, productivity and yield of 80–120 g L^−1^, 2–6 g L^−1^h^−1^ and 80–95 %, respectively. Few of these strains, however, have the ability to ferment xylose into D-lactic acid with a desired titer, yield and rate [3, 23, 24, 30, 31]. Furthermore, none, if any, of these strains have demonstrated the ability to co-metabolize both glucose and xylose for enhanced D-lactic acid fermentation.

Like most bacteria, *E. coli* has a preference for glucose over other sugars for energy [7, 13]. Whenever glucose and other sugars are available, it will use glucose first, then other sugars only if glucose is completely consumed. This phenomena is often called the glucose effect or catabolite repression. The sequential use of glucose and xylose often results in delayed and incomplete use of xylose for lactic acid fermentation using sugar mixtures [8, 23]. Eliminating the glucose effect is needed to allow co-utilization of glucose and xylose for improved D-lactic acid production using cellulosic substrates [11, 14].

In this study, we report reengineering *E. coli* WL204 (Δ*frdBC* Δ*ldhA* Δ*ackA* Δ*pflB* Δ*pdhR ::pflBp6-acEF-lpd* Δ*mgsA* Δ*adhE*, Δ*ldhA*::*ldhL*) [30] for D-lactic acid production by 1) replacing the L-lactate dehydrogenase gene (*ldhL*) with a D-lactate dehydrogenase gene (*ldhA*); 2) eliminating catabolite repression via deletion of the *ptsG* gene that encodes for IIBC^glc^, a major enzyme of the glucose PTS system; 3) adaptive evolution in screw-cap tubes for improved cell growth with glucose as the sole substrate. The resulting strain, *E. coli* JH15, is able to co-utilize both glucose and xylose for enhanced D-lactic acid production.

## Results and discussion

### Engineering of *E. coli *JH13 for optically pure D-lactic acid production

**Fig. 1:**
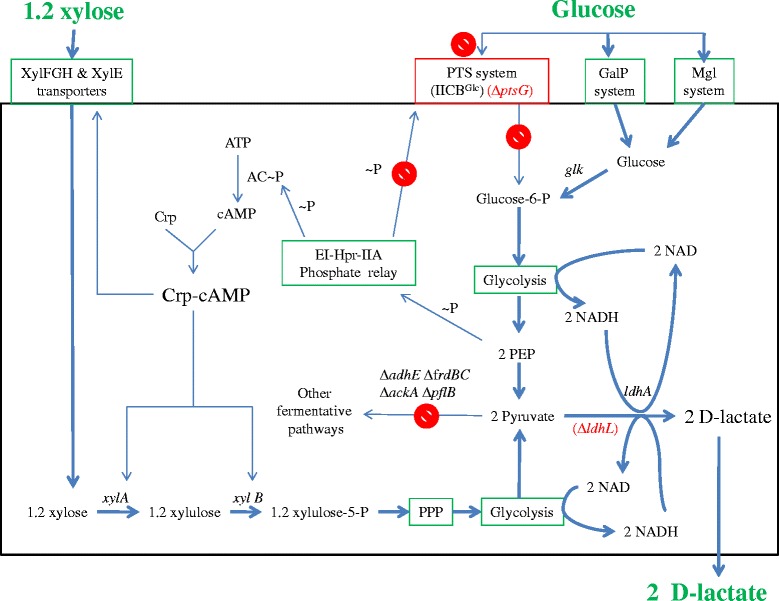
Engineering an *E. coli* strain devoid of the glucose effect for D-lactic acid production from a mixture of glucose and xylose. Genes encoding important enzymes are indicated by italics. The relevant genes/enzymes are: *ldhL*, L-lactate dehydrogenase; *ldhA*, D-lactate dehydrogenase; *adhE*, alcohol dehydrogenase; *ackA*, acetate kinase; *pflB*, pyruvate formate lyase; *frdABCD*, fumarate reductase; *glk*, glucokinase; *xylA*, xylose isomerase; *xylB*, xylulokinase; *xylFGH*, xylose ABC transporter; *xylE*, xylose/proton symporter; *ptsG*, subunit of glucose PTS permease; EI-Hpr-IIA, phosphoenolpyruvate-protein phosphotransferase system; AC-P, adenylate cyclase; ~P, high-energy phosphate from a phosphorylated compound; Crp, cAMP receptor protein; Crp-cAMP, transcriptional dual regulator; GalP, galactose transporter; Mgl, galactose transporter. Symbols: the stop and delta (∆) signs indicated the relevant genes (*adhE*, *frdBC*, *ackA*, *pflB*, *ptsG*, *ldhL*) were deleted

*E. coli* WL204, a xylose fermenting homo-L-lactate producing strain previously engineered from *E. coli* B [30], was used as the starting strain (Fig. 1). Although a one-step gene replacement (*ldhL* (encodes for L-lactate dehydrogenase) replaced by *ldhA* (encodes for D-lactate dehydrogenase)) in WL204 would allow the resulting strain produce D-lactic acid, the selection process will be complicated because the correct replacement (of *ldhL* by *ldhA*) will have to rely on the fermentation and/or sequence results. To make a simple selection, a two-step strategy was used to allow plate selection. L-lactate production was first eliminated through deletion of the recombinant L-lactate dehydrogenase gene (*ldhL*) using an *ldhA*’-FRT-*kan*-FRT-*ldhA*’ DNA fragment. The *ldhL* gene was replaced by the kanamycin resistance marker (*kan*) through double homologous recombination facilitated by the λ red recombination system [5, 22, 30]. The resulting strain, JH12 (∆*ldhL*:: FRT-*kan-*FRT), lost L-lactic acid production as well as anaerobic cell growth due to the loss of L-lactate dehydrogenase, blocking NADH oxidation (Fig. 1). Nevertheless, it grows well aerobically on either glucose or xylose plates.

To enable D-lactic acid production as well as anaerobic growth, a D-lactate dehydrogenase is needed to reestablish a balanced NADH/NAD redox (1 glucose or 1.2 xylose + 2 NAD (glycolysis) = > 2 pyruvate + 2 NADH (D-lactate dehydrogenase) = > 2 D-lactate + 2 NAD) (Fig. 1). To this end, a D-lactate dehydrogenase gene (*ldhA*) was amplified by PCR using the chromosomal DNA of *E. coli* B as the template. The amplified DNA fragment contained the *ldhA* coding region flanked by 1460 bp upstream of the start codon and 600 bp downstream of the stop codon. This ~3 kb DNA fragment was transformed into JH12 (pKD46) through electroporation. Through double homologous recombination, facilitated by λ red recombinase , this DNA fragment was integrated into JH12 chromosome, replacing the kanamycin marker. The recombinant strain was selected through regaining anaerobic cell growth in screw-cap tubes and was tested by loss of kanamycin resistant. After curing the temperature sensitive plasmid pKD46 at 42 °C, the resulting strain was designated JH13 (∆FRT-*kan-*FRT::*ldhA*).

**Fig. 2:**
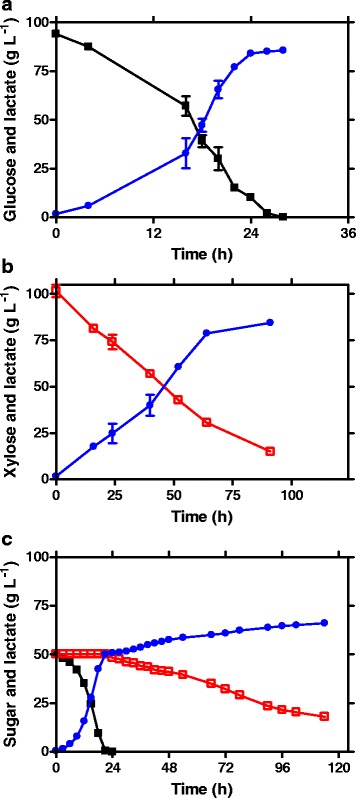
Fermentative production of D-lactic acid by strain JH13, **a** 100 g L^−1^ glucose; **b** 100 g L^−1^ xylose; **c** 100 g L^−1^ mixture of glucose and xylose. Symbols: filled square, glucose; open square, xylose; filled circle, D-lactic acid. Each data point represents the average of two or more replicates. The error bar represents the standard deviations

JH13 was evaluated for D-lactic acid production using 100 g L^−1^ of glucose, xylose or a mixture of glucose/xylose. As shown in Fig. 2 and Table 1, glucose was converted into D-lactic acid in 28 h, achieving a titer of 85 g L^−1^ and a 90 % glucose-to-D-lactic acid conversion yield. A similar titer (84 g L^−1^) and yield (83 %) was obtained in xylose fermentation (Fig. 2b). However, the maximal and average D-lactic acid productivity achieved in xylose fermentation (1.63 g L^−1^h^−1^, 0.93 g L^−1^h^−1^, respectively) was ~1/3 of that obtained in glucose fermentation (5.44 g L^−1^h^−1^, 3.04 g L^−1^h^−1^, respectively). The lower rate of xylose consumption and D-lactic acid productivity is mostly attributed to its lower ATP yield of xylose catabolism (0.67 net ATP per glucose equivalent) compared to glucose catabolism (2 net ATP per glucose) [9]. Nevertheless, with an extended fermentation time, complete xylose fermentation was achieved and resulted in a similar D-lactic acid titer as that of glucose fermentation.

**Table 1:** Fermentations summary by *E. coli* JH13 and JH15

Strain	Substrate (100 g L^−1^)	Fermentation Time (h)	Sugar consumption (g L^−1^ h^−1^)^a^						Lactic acid production (g L^−1^ h^−1^)^b^			
			Glucose		Xylose		Total (glu + xyl)		Titer (g L^−1^)	Yield (%)	Vol. productivity (g L^−1^ h^−1^)	
			Max	Average	Max	Average	Max	Average			Max	Average
JH13	Glucose	28	5.88	3.36					85	90	5.44	3.04
	Xylose	91			1.65	0.95			84	83	1.63	0.93
	Glu + Xyl	114	3.42	2.08	0.6	0.39	n/a^c^	0.71	66	80	n/a^3)^	0.58
JH15	Glucose	36	2.88	2.69					88	91	3.75	2.44
	Xylose	120			1.38	0.8			81	83	1.13	0.67
	Glu + Xyl	96	1.89	1.28	0.95	0.52	2.2	1.04	83	83	2.53	0.86

^a^The maximum sugar consumption was calculated from the fastest 8 h, or longer, fermentation period. The average sugar consumption was calculated from the active fermentation period (0 h to the end of fermentation)

^b^The yield was calculated based on lactic acid produced over sugar metabolized. The maximum lactic acid productivity was calculated from the fastest 8 h, or longer, fermentation period. The average lactic acid productivity was calculated from the active production period (0 h to the end of fermentation)

^c^n/a is not applicable (since glucose and xylose was used separately in JH13, the maximum total sugar consumption and maximum lactic acid productivity would be derived from glucose fermentation only)

When tested in a 100 g L^−1^ mixture of glucose (50 g L^−1^) and xylose (50 g L^−1^), as expected, a sequential use of glucose and xylose was observed due to catabolite repression. As shown in Fig. 2c, glucose was first catabolized into D-lactic acid. After 24 h, cells started to ferment xylose once the glucose was completely consumed. This delayed xylose utilization, as well as the lower consumption rate of xylose (0.39 g L^−1^h^−1^) compared to glucose (2.08 g L^−1^h^−1^), resulted in an incomplete xylose utilization, a lower D-lactic acid titer (66 g L^−1^), and lower productivity (0.58 g L^−1^h^−1^) compared to that achieved in 100 g L^−1^ glucose fermentation (85 g L^−1^, 3.04 g L^−1^h^−1^, respectively) and xylose fermentation (84 g L^−1^, 0.93 g L^−1^h^−1^, respectively).

**Fig. 3:**
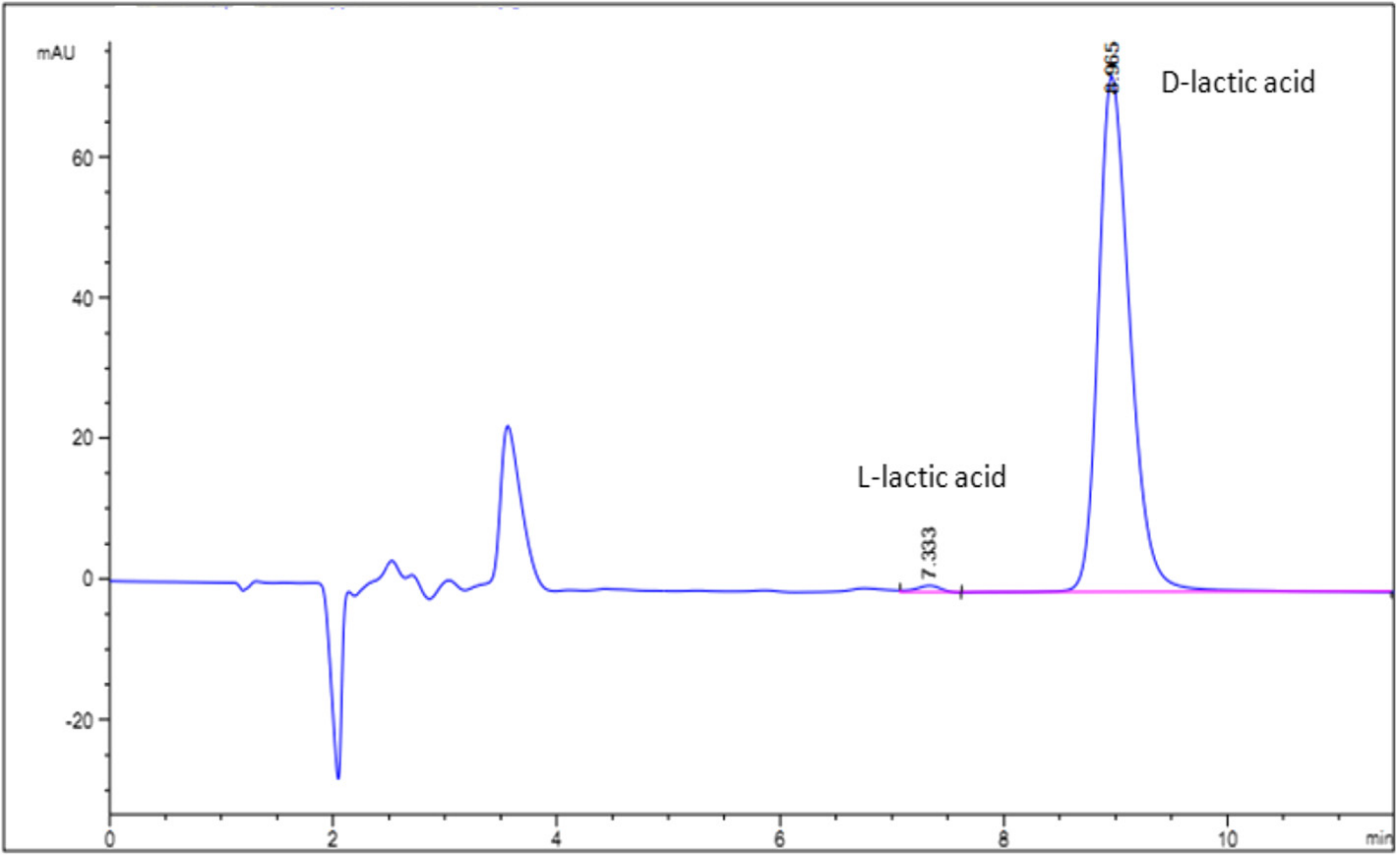
HPLC analysis of the optical purity of D-lactic acid produced by strain JH13 (and/or JH15). No L-lactic acid peak was detected in the fermentation broth of JH13 (and/or JH15). The L-lactic acid peak detected was the external L-lactic acid intentionally added into the fermentation broth (L-lactic acid added represents 0.6 % of the total lactic acid in the broth)

When tested for optical purity of the fermentation products, regardless of whether the fermentation substrate was glucose, xylose or a mixture of glucose/xylose, only a D-lactic acid peak was detected in the fermentation broth of JH13. L-lactic acid, if present, was under the detectable level of HPLC analysis using a chiral column and the standard D- and L-lactic acid isomers. Nevertheless, an L-lactic acid peak was detected if external L-lactic acid (represented 0.6 % of the total lactic acid) was intentionally added into the broth (Fig. 3).

### Eliminating the glucose effect/catabolite repression

Catabolite repression in *E. coli* has been elucidated at a genetic level by others (Fig. 1) [7, 11, 13]. Briefly, the transcription of *xylFGH*, *xylE*, and *xylAB* genes encoding for xylose transporters and catabolic enzymes are positively regulated by the Crp-cAMP complex. The formation of this activator is limited by the availability of cAMP. The biosynthesis of cAMP is catalyzed by adenylate cyclase (AC ~ P), which is activated by phosphorylation via the PEP-EI-Hpr-IIA phosphate relay. When glucose is present, AC is outcompeted by IICB^glc^ (a glucose transport enzyme encoded by *ptsG*) for phosphorylation. Most, if not all, phosphate from the PEP-EI-Hpr-IIA phosphate relay is used for IICB^glc^ phopsphorylation, resulting in active glucose transport (Fig. 1) rather than activation of AC. Consequently, cAMP and Crp-cAMP are not formed, resulting in no activation of the xylose utilization genes. When glucose is completely used, AC is activated by the PEP-EI-Hpr-IIA phosphate relay, cAMP is made, and Crp-cAMP is formed to turn on the xylose utilization genes.

To eliminate catabolite repression and allow co-utilization of glucose and xylose for improved D-lactic acid production, the *ptsG* gene encoding for the IICB^glc^ was deleted from *E. coli* JH13 using a *ptsG*’-FRT-*kan*-FRT-*ptsG*’ DNA fragment, resulting in strain JH14 (∆*ptsG*::FRT-*kan-*FRT). When tested by fermentation of glucose and xylose mixtures, JH14 co-utilized both glucose and xylose, suggesting that catabolite repression was successfully eliminated. Nevertheless, in glucose, JH14 grew slower and achieved a lower final OD_600_ than JH13 (data not shown).

**Fig. 4:**
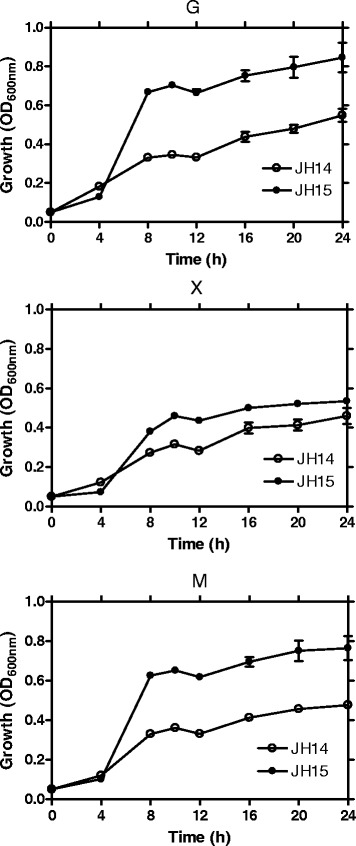
Metabolically evolved strain regained improved anaerobic cell growth. G) 20 g L^−1^ glucose NBS medium; X) 20 g L^−1^ xylose NBS medium; M) 20 g L^−1^ mixed glucose and xylose NBS medium. Symbols: open circle, JH14, initially engineered strain devoid of the glucose effect; filled circle, JH15, metabolically evolved strain from JH14. Each data point represents the average of three replicates. The error bar represents the standard deviations

The PTS system enables *E. coli* to use glucose as a preferred energy source because it allows glucose transport and phosphorylation to take place in one step. The resulting glucose-6-phosphate can enter glycolysis directly without further phosphorylation by glucokinase. With the disruption of the PTS system by the *ptsG* deletion, JH14 will have to rely on an alternative system such as the galactose transporter, GalP and Mgl, for glucose transport. However, the GalP and Mgl system is much less efficient than the PTS for glucose transport, resulting in slower anaerobic cell growth and glucose fermentation (Fig. 4g).

### Evolutionary engineering for improved cell growth and glucose utilization

To compensate for the loss of the PTS system, JH14 needs an improved alternative glucose transporter. Cloning a heterologous glucose transporter may or may not work for our strain improvement because most bacteria use PTS system for glucose transport which may reintroduce the glucose effect into the strain. Other non-PTS system may still be inefficient for glucose transport. Therefore, an evolutionary engineering approach was used for strain improvement. To this end, JH14 was metabolically evolved for three months via selection of improved cell growth using screw-cap tubes containing glucose as the sole energy source. In the end, a single colony was selected from the evolved culture and was designated as strain JH15. The cell growth of the evolved JH15 strain was compared to that of JH14 in screw-cap tubes containing glucose and/or xylose as the energy source. As shown in Fig. 4, a lag growth phase of ~4 h, followed by a logarithmic growth phase of ~4 h (4–8 h) was observed for both strains regardless of whether glucose or xylose was used. During the logarithmic phase, however, the growth rate and cell mass achieved by the evolved strain, JH15 (0.409 h^−1^, 0.318 g L^−1^, respectively), was at least twice that obtained by JH14 (0.148 h^−1^, 0.157 g L^−1^, respectively) when glucose was used as the energy source (Fig. 4G). A similar pattern was observed when xylose was used as the energy source (Fig. 4X). The evolved strain JH15 grew better than JH14, the growth difference of the two strains, however, was not as significant as that observed in glucose conditions. This result led us speculate that the improved cell growth of JH15 was mostly attributed to its improved glucose uptake via the GalP and/or Mgl system (Fig. 1).

### Co-utilization of glucose and xylose enhanced D-lactic acid production

**Fig. 5:**
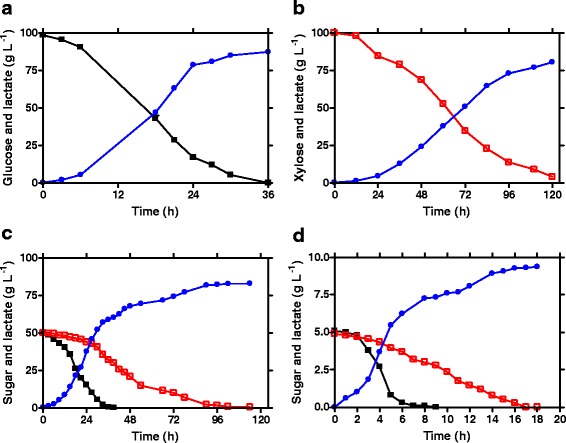
Fermentative production of D-lactic acid by strain JH15. **a** 100 g L^−1^ glucose; **b** 100 g L^−1^ xylose; **c** 100 g L^−1^ mixture of glucose and xylose; **d** 10 g L^−1^ mixture of glucose and xylose. Symbols: filled square, glucose; open square, xylose; filled circle, D-lactic acid. Each data point represents the average of two or more replicates. The error bar represents the standard deviations

It was expected that JH15 would regain the ability of efficient D-lactic acid fermentation using glucose and xylose. To test this hypothesis, JH13 and JH15 were compared for D-lactic acid fermentation using 100 g L^−1^ of: 1) glucose; 2) xylose; and 3) a mixture of glucose (50 g L^−1^) and xylose (50 g L^−1^). As the results demonstrated in Fig. 5 and Table 1, the evolved strain JH15 regained the fermentative ability for D-lactic acid production. In glucose fermentation, JH15 achieved a D-lactic acid titer of 88 g L^−1^ and a yield of 91 % (Fig. 5a), which is comparable to results obtained by JH13 (85 g L^−1^, and 90 %, respectively; Fig. 2a). However, 36 h was needed for JH15 to complete the 100 g L^−1^ glucose fermentation; 8 h longer than the fermentation time needed by JH13 (28 h). The longer fermentation resulted in a 20 % lower productivity (2.44 g L^−1^h^−1^ vs 3.04 g L^−1^h^−1^). This result suggested that the improved alternative glucose transporter in JH15 is not as effective as the original PTS glucose transporter in JH13. In xylose fermentation, the performance was similar for both strains (Figs. 2b and 5b ), although JH15 had a 12 h longer lag phase than JH13.

A significant improvement of D-lactic acid production was achieved by JH15 in mixed glucose and xylose fermentation (Fig. 5c), compared to that of JH13 (Fig. 2c). Both glucose and xylose were co-consumed by JH15, allowing a 32 % higher total sugar consumption rate (1.04 g L^−1^h^−1^) than that of sequential sugar utilization by JH13 (0.71 g L^−1^h^−1^). Consequently, the faster sugar consumption enabled JH15 to achieve a 33 % higher productivity (0.86 g L^−1^h^−1^) as well as a 20 % higher D-lactic acid titer (83 g L^−1^) compared to that obtained by JH13 (0.58 g L^−1^h^−1^, 66 g L^−1^, respectively). Furthermore, the 83 g L^−1^ D-lactic acid titer achieved in mixed sugar fermentation was comparable to that obtained in glucose (88 g L^−1^) or xylose (81 g L^−1^) fermentations.

Although both glucose and xylose were co-consumed (Fig. 5c), JH15 had a lower xylose consumption rate (0.25 g L^−1^h^−1^) when glucose concentration was greater than 15 g L^−1^ (0–24 h) compared to that (1.17 g L-^1^h^−1^) when glucose was less than 15 g L^−1^ (24–36 h). Nevertheless, the total sugar consumption rate (1.71 g L^−1^h^−1^ vs 2.41 g L^−1^h^−1^) and lactate production rate (1.6 g L^−1^h^−1^ vs 1.75 g L^−1^h^−1^) were similar regardless of glucose concentration or xylose consumption rate during these fermentation periods (0-24 h and 24-36 h). These observations suggested that at a given lactate production rate, when both glucose and xylose concentrations were high (50 g L^−1^) at early time, xylose was outcompeted by glucose due to its lower net energy output (0.67 ATP per glucose equivalent) than glucose (2 ATP), resulting in a lower xylose consumption rate (0-24 h). After 24 h fermentation, however, more xylose (44 g L^−1^) was available than glucose (15 g L^−1^). To achieve an equivalent lactate production rate, more xylose would be used to compensate the less available glucose, resulting in higher xylose consumption rate (24–36 h).

The observed different xylose consumption rate with different glucose availability led us to test whether both glucose and xylose were co-consumed by JH15 at lower sugar concentration. As the results shown in Fig. 5d, JH15 was able to co-metabolize both glucose and xylose with a starting sugar concentration as low as 5 g L^−1^.

**Fig. 6:**
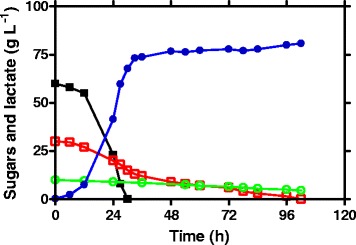
Fermentative production of D-lactic acid in a simulated biomass hydrolysate by strain JH15. Symbols: filled square, glucose; open square, xylose; open circle, L-arabinose; filled circle, D-lactic acid. Each data point represents the average of two replicates

Furthermore, JH15 was tested for co-fermentation of glucose (60 g L^−1^), xylose (30 g L^−1^) and L-arabinose (10 g L^−1^) in a simulated cellulosic biomass hydrolysate. As shown in Fig. 6, three sugars were co-metabolized, producing 81 g L^−1^ of D-lactic acid. Nevertheless, there was approximately 4 g L^−1^ of L-arabinose were unutilized although both glucose and xylose were completely consumed.

## Conclusions

An *E. coli* strain was engineered and adaptively evolved for improved production of optically pure D-lactic acid from mixed glucose and xylose substrates (Fig. 3). The evolved strain, JH15, is able to co-metabolize both glucose and xylose, resulting in an increase in the sugar consumption rate by 46 %, productivity by 48 %, and a D-lactic acid titer by 26 % compared to that of sequential utilization of glucose and xylose by JH13. These results demonstrated that JH15 has the potential for D-lactic acid production using cellulosic substrates which contain both glucose and xylose. Nevertheless, further improvements in productivity are needed for practical applications.

## Methods

### Bacterial strains, plasmids and media

The bacterial strains, plasmids, and primers used in this study are listed in Table 2. During strain construction, cells were grown in Luria–Bertani broth (g L^−1^: tryptone 10, yeast extract 5, and NaCl 5) containing glucose or xylose (g L^−1^: 20) or on LB plates (g L^−1^: agar 20, xylose 20). Ampicillin or kanamycin (50 μg ml^−1^) was added into the medium as needed. The engineered strain was maintained on mineral salts medium (NBS medium (g L^−1^): KH_2_PO_4_, 3.5; K_2_HPO_4_, 5.0; (NH_4_)_2_HPO_4_, 3.5; MgSO_4_:7H_2_O, 0.25; CaCl_2_:2H_2_O, 0.015; thiamine, 0.0005; and 1 mL of trace metal stock) [31, 32]. Fermentations were carried out in LB broth or modified mineral salts medium (NBS + 2.0 g L^−1^yeast extract).

**Table 2:** *E. coli* strains, plasmids and primers used in this study

Strains	Relevant characteristics	Sources
B	Wild type	ATCC
WL204	*E. coli* B, Δ*frdBC* Δ*ldhA* Δ*ackA* Δ*pflB* Δ*pdhR ::pflBp6-acEF-lpd* Δ*mgsA* Δ*adhE*, Δ*ldhA*::*ldhL*, metabolically evolved in xylose with improved anaerobic growth	[30]
JH12	WL204, Δ*ldhL::*FRT-*kan*-FRT (chromosomal insertion of *kan* marker to replace *ldhL* gene), lost anaerobic cell growth, kanamycin positive	This study
JH13	JH12, ΔFRT-*kan*-FRT::*ldhA* (chromosomal insertion of *ldhA* to replace *kan* marker), regaining anaerobic cell growth, kanamycin sensitive	This study
JH14	JH13, Δ*ptsG::*FRT-*kan*-FRT, slow glucose utilization due to disruption of the major glucose transporter system (PTS system)	This study
JH15	JH14, metabolically evolved in glucose with improved glucose uptake (probably through alternative glucose transporter)	This study
Plasmids		
pKD4	FRT-*kan*-FRT cassette	[5]
pKD46	*bla*, red recombinase, temperature-dependent replication	[5]
pFT-A	*bla*, *flp*, temperature-dependent replication	[22]
Primers		
Deletion *ldhL*-P1	TGTTTCGCTTCACCGGTCAGCTGTGTGTAGGCTGGAGATGCTTC ^a^	This study
Deletion *ldhL*-P2	TCGCTAATGGTGTTATCGAGTTAGCCATATGAATATCCTCCTTAG ^a^	This study
Cloning *ldhA*-P1	TGCAGCACGA AATCGCCCAG TTCAT	This study
Cloning *ldhA*-P2	TGTGTGCATTACCCAACGGCAAACG	This study
Deletion *ptsG*-P1	ATGTTTAAGAATGCATTTGCTAACCGTGTGTAGGCTGGAGATGCTTC ^a^	This study
Deletion *ptsG*-P2	TTAGTGGTTACGGATGTACTCATCCAAGCCATATGAATATCCTCCTTAG ^a^	This study

^a^The underlined sequence is homologous to the flanked sequence of FRT-kan-FRT cassette in pKD4

### Genetic methods

Transformation, electroporation, PCR, and DNA fragment analyses were conducted using standard methods. Chromosomal gene deletions and integrations were carried out using previously described λ red homologous recombination procedures [5, 22, 31]. The gene deletions and integrations were verified by using appropriate antibiotic markers, PCR fragment analysis, and HPLC analysis of fermentation products.

### Evolutionary engineering

The initial strain devoid of catabolite repression, JH14, was inoculated into a 250 ml flask containing 50 ml NBS broth with 5 % glucose, and was incubated for 16 h (37 °C, 150 rpm). 100 μl cells from the flask was inoculated into a 10 ml screw-cap tube containing ~9.5 ml NBS broth with 2 % glucose. Upon sealing the cap, the tube culture was incubated at 37 °C (50 rpm) for 48 h. 100 μl culture from the tube was transferred into another tube containing fresh medium and incubated again for 48 h. This transfer selection was repeated for two month. A 24 h transfer interval was then applied for an additional month to further enrich faster growing cells. At the end of evolutionary engineering, the culture was streaked out for isolation of single colonies. After testing their growth in screw-cap tubes with glucose as the sole energy source, one of these colonies was designated JH15.

### Fermentations

Seed cultures: fresh colonies from NBS plates were inoculated into 1000 ml flasks containing 400 ml modified NBS medium with 2 % (w/v) xylose, incubated at 37 °C, and 200 rpm for 12–16 h to achieve an OD_600nm_ of ~1.5 (The conversion factor DCW/OD is 0.35 g L^−1^).

Fermentations: seed cultures were inoculated for a starting OD of 0.1 (0.035 g L^−1^ cell dry weight) into a 7 L fermenter (Sartorius Stedim Biotech GmbH 37070, Germany) containing 4 L modified NBS medium with 100 g L^−1^ of glucose or xylose or a mixture of equal amounts of glucose and xylose. The fermentation was carried out at 37 °C, 200 rpm. The fermentation was pH controlled by automatic addition of 6 N Ca(OH)_2_. Fermentations were terminated when no base was needed to maintain the controlling pH. Fermentation broth was withdrawn (1.5 ml) periodically and analyzed by HPLC for sugar consumption and lactic acid production. All fermentations had 2 or more repeats.

### Analysis

Cell growth was estimated from the optical density at 600 nm (0.35 g dry cell weigh per OD_600_). Fermentation samples were centrifuged at 8,000 rpm for 10 min. The supernatant was then treated with H_2_SO_4_ and centrifuged again to remove the precipitate (CaSO_4_). The supernatant was then filtered through a 0.22 μm membrane and analyzed for sugar and organic acid concentrations by an Agilent HPLC (column, BioRad HPX 87H; temperature, 35 °C; flow rate, 0.5 ml min^−1^; mobile phase, 4 mM H_2_SO_4_). Optical purity was determined by HPLC using a chiral column (EC 250/4 NUCLEOSIL Chiral-1, Germany) (HPLC condition: 35 °C, 0.5 ml min^−1^ of 0.2 mM CuSO_4_ as the mobile phase).

## Abbreviations

PLA: polylactic acid
PDLA: poly-D(−)-lactic acid
PLLA: poly-L(+)-lactic acid
SC-PLA: stereo-complex polylactic acid
*frd*: fumarate reductase
*ldhA*: D-lactate dehydrogenase
*ldhL*: L-lactate dehydrogenase
*ackA*: acetate kinase
*pflB*: pyruvate formate lyase
*pdhR*: repressor of pyruvate dehydrogenase operon
*pflB*p6: promoter 6 of pyruvate formate lyase
*aceEF-lpd*: pyruvate dehydrogenase operon
*mgsA*: methylglyoxal synthase
*adhE*: alcohol dehydrogenase
*ptsG*: subunit of glucose PTS permease
EI-Hpr-IIABC: phosphoenolpyruvate-protein phosphotransferase system
NAD: nicotinamide adenine dinucleotide
NADH: reduced form of nicotinamide adenine dinucleotide
FRT: flipase recognition target
pKD46: plasmid containing the red recombinase system
ATP: adenosine triphosphate
HPLC: high pressure liquid chromatography
*glk*: glucokinase
*xylA*: xylose isomerase
*xylB*: xylulokinase
*xylFGH*: xylose ABC transporter
*xylE*: xylose/proton symporter
AC-P: adenylate cyclase
~P: high-energy phosphate from a phosphorylated compound
Crp: cAMP receptor protein
Crp-cAMP: transcriptional dual regulator
GalP: galactose transporter
Mgl: galactose transporter, PCR, polymerase chain reaction
OD_600_: optical density at 600 nm
DCW: dry cell weight
